# COVID-19 transmission dynamics and the impact of vaccination: modelling, analysis and simulations

**DOI:** 10.1098/rsos.221656

**Published:** 2023-07-26

**Authors:** Joseph Malinzi, Victor Ogesa Juma, Chinwendu Emilian Madubueze, John Mwaonanji, Godwin Nwachukwu Nkem, Elias Mwakilama, Tinashe Victor Mupedza, Vincent Nandwa Chiteri, Emmanuel Afolabi Bakare, Isabel Linda-Zulu Moyo, Eduard Campillo-Funollet, Farai Nyabadza, Anotida Madzvamuse

**Affiliations:** ^1^ Faculty of Science and Engineering, Department of Mathematics, University of Eswatini, Private Bag 4, Kwaluseni, Swaziland; ^2^ Institute of Systems Science, Durban University of Technology, Durban 4000, South Africa; ^3^ Multiscale in Mechanical and Biological Engineering (M2BE), Instituto de Investigación en Ingeniería de Aragón (I3A), University of Zaragoza, 50018 Zaragoza, Spain; ^4^ Department of Mathematics, Federal University of Agriculture, Makurdi, Nigeria; ^5^ Department of Mathematics and Statistics, York University, Toronto, Canada; ^6^ Department of Mathematical Sciences, Malawi University of Business and Applied Sciences, Blantyre, Malawi; ^7^ Department of Mathematics, University of Ibadan, Ibadan, Nigeria; ^8^ Department of Pure and Applied Mathematics, Jomo Kenyatta University of Agriculture and Technology, Nairobi, Kenya; ^9^ Department of Mathematics & Computational Sciences, University of Zimbabwe, Box MP167 Mount Pleasant, Harare, Zimbabwe; ^10^ Department of Mathematics, University of Nairobi, Nairobi, Kenya; ^11^ International Centre for Applied Mathematical Modelling and Data Analytics, Federal University Oye-Ekiti, Ekiti State, Nigeria; ^12^ Department of Mathematics, Federal University Oye-Ekiti, Ekiti State, Nigeria; ^13^ Department of Mathematics and Statistics, Lancaster University, Lancaster LA1 4YR, UK; ^14^ Department of Mathematics and Applied Mathematics, University of Johannesburg, Auckland Park 2006, South Africa; ^15^ Mathematics Department, Room 121, Mathematics Building, University of British Columbia, 1984 Mathematics Road, Vancouver, BC, Canada V6T 1Z2; ^16^ School of Mathematical and Physical Sciences, Department of Mathematics, University of Sussex, Brighton BN1 9QH, UK

**Keywords:** COVID-19, vaccinations, mathematical modelling, parameter estimation, sensitivity analysis, bifurcation analysis

## Abstract

Despite the lifting of COVID-19 restrictions, the COVID-19 pandemic and its effects remain a global challenge including the sub-Saharan Africa (SSA) region. Knowledge of the COVID-19 dynamics and its potential trends amidst variations in COVID-19 vaccine coverage is therefore crucial for policy makers in the SSA region where vaccine uptake is generally lower than in high-income countries. Using a compartmental epidemiological model, this study aims to forecast the potential COVID-19 trends and determine how long a wave could be, taking into consideration the current vaccination rates. The model is calibrated using South African reported data for the first four waves of COVID-19, and the data for the fifth wave are used to test the validity of the model forecast. The model is qualitatively analysed by determining equilibria and their stability, calculating the basic reproduction number $R_{0}$ and investigating the local and global sensitivity analysis with respect to $R_{0}$. The impact of vaccination and control interventions are investigated via a series of numerical simulations. Based on the fitted data and simulations, we observed that massive vaccination would only be beneficial (deaths averting) if a highly effective vaccine is used, particularly in combination with non-pharmaceutical interventions. Furthermore, our forecasts demonstrate that increased vaccination coverage in SSA increases population immunity leading to low daily infection numbers in potential future waves. Our findings could be helpful in guiding policy makers and governments in designing vaccination strategies and the implementation of other COVID-19 mitigation strategies.

## 1. Introduction

Many countries are still struggling with the effects of the coronavirus SARS-CoV-2 and the associated disease COVID-19, that was long declared a pandemic by the World Health Organization (WHO). Despite the widespread global public health initiatives, for example social distancing, wearing face masks, hand washing with clean water and soap or using alcohol-based hand sanitizer, the pandemic continued to pose a serious threat with unmatched impact to public health. As of 18 October 2022, nearly 625 million infected cases and 6.57 million COVID-19-related deaths were reported worldwide [1]. Nonetheless, COVID-19 vaccination programmes, as a means of preventing an individual from catching the infection, have been implemented on a global scale, with several vaccines approved [2]. However, several countries especially in Africa have registered low uptake of COVID-19 vaccine [3]. Moreover, challenges still remain, ranging from securing the vaccines to their distribution. The control measures that have, in the past, been implemented have led to devastating effects on individual and global economies including the decrease in industrial production which has in a way contributed to inflation and sharp rise in commodity prices [4,5].

Vaccination is a control measure that can be taken without substantially negatively impacting the economy, but a number of questions need to be answered to realize the maximum benefits. Firstly, determining the possible number of waves a country could possibly experience, determining what percentage of a population should be vaccinated in order to curtail the disease, determining the overall effect of vaccination in subsequent waves and forecasting the outcomes of infections with several vaccination rates. Furthermore, a recovered individual is not immune to other variants and waning of immunity towards COVID-19 has been reported [6].

Mathematical models have proven effective in describing the dynamics of numerous epidemics: by estimating the reproduction numbers, estimating herd immunity thresholds and ascertaining the period the epidemic would take to reach its peak or go to extinction. For example, numerous studies [7–24] have characterized different aspects of COVID-19 dynamics, ranging from determining the epidemic curves [10,11], investigating the efficacy of the control measures [8,11], to assessing the impact of vaccination [25]. Several excellent mathematical studies have been carried out on COVID-19 in southern Africa. We briefly review some studies that are closely related to the objectives of our study in the context of South Africa.

Nyabadza *et al.* [7] proposed a susceptible–exposed–infected–recovered (SEIR) model to investigate the impact of social distancing on the transmission dynamics of COVID-19 in South Africa in which the predictions that were made were close to what was observed in South Africa at the beginning of the epidemic. They fitted their model to data from cumulative number of reported infected cases in South Africa. The results of their study depicted a continued increase in the number of infected cases even during the first lock down period. Their analysis further showed that increasing the level of social distancing by 2% would reduce the number of cumulative cases by 18% whereas a reduction, of social distancing, by the same margin would lead to a 23% increase in the number of cases. Anguelov *et al.* [10] investigated the role played by asymptomatic cases in the disease propagation, premised on the notion that the asymptomatic cases for COVID-19 are rarely recorded, thus representing an unknown effect that can be significant in determining the eventual long-term dynamics of the disease. They argue that the dynamics are greatly influenced by the population’s acquired immunity which is built through both symptomatic and asymptomatic infections. They assert that it is imperative to determine the ratio of symptomatic to asymptomatic cases in order to determine the size and peak of infections such that plausible control actions can be suggested. Garba *et al.* [23] used a compartmental model to analyse the transmission dynamics of the disease in South Africa using available data. Analysis of the model revealed that its associated continuum of disease-free equilibria was then globally asymptotically stable whenever the control reproduction number was less than unity. The study suggested that the disease eventually dies out, particularly if control measures are implemented early and for a sustainable period of time [23].

Mathematical modelling of COVID-19 has, however, moved away from simply determining epidemic curves and/or determining effectiveness of the different control measures to investigating the complexity of the disease. For example, current studies suggest determining wave periods and peaks, herd immunity, assessing the impact of vaccination and determining the best vaccine [20–22,25–27]. The concept of herd immunity, in relation to COVID-19, may be somewhat illusive, as it may not be possible to obtain such a threshold where, by vaccinating a certain proportion of the population would indicate that COVID-19 is put under control [28]. This is mainly due to the fact that COVID-19 has several variants and by vaccinating against one does not guarantee total immunity against the others. Nonetheless, it should still be of great importance either to calculate a similar threshold or to rather estimate the percentage of a certain population that would need to be vaccinated (against which variants or generally) in order to lessen the disease effects (with minimal costs). Here, we strive to determine the current COVID-19 dynamics after incorporating vaccination and then forecast the possible future trends if such vaccination rates were to be maintained or if the rates were increased. The study also forecasts future trends for different vaccination efficacy.

Generally, herd immunity is a key concept for epidemic control. It states that only a proportion of a population needs to be immune (through overcoming natural infection or through vaccination) to an infectious agent for it to stop generating large outbreaks. In studies [26,29], a key question with regard to COVID-19 pandemic was how and when herd immunity can be achieved and at what cost. They found that there was little evidence to suggest that the spread of SARS-CoV-2 might stop naturally before at least 50% of the population had become immune. They also found that the cost of reaching herd immunity through natural infection would be very high especially in the absence of improved patient management and without optimal shielding of individuals at risk of severe complications. Another study [27] found that in the absence of a vaccine, building up SARS-CoV-2 herd immunity through natural infection is theoretically possible. However, there was no straightforward, ethical path to accomplish that, as the societal consequences of achieving it are devastating.

In Weitz *et al.* [13], a novel COVID-19 intervention (shield immunity) is proposed. The measure uses serological tests to identify and strategically deploy recovered individuals that could have developed protective antibodies to the pathogen. A mathematical model that uses the SEIR framework was extended to incorporate serological testing. Their model simulations indicate that when serological testing is employed such that seropositive individuals are purposefully placed, a substantial decrease in the number of infected, hospitalized and mortality cases is observed. This study, however, ignored the existence of several variants which would make the use of shield immunity less effective as serological individuals would only give protection for a particular variant or only a few and certainly not all. Nonetheless, studies like these have suggested better alternatives to the control of COVID-19 other than the traditional mitigation and suppression measures.

The COVID-19 pandemic caused multiple waves of cases and deaths in the USA. Despite the introduction of vaccination, multiple waves continued to take their toll on the population [30]. In order to examine the effects of the vaccination across US states, the classic SEIR model was used. It was found that vaccination averted death but could not prevent subsequent waves occurring due to the wild strain, the Alpha (B.1.1.7) and the Delta (B.1.617.2) variants of SARS-CoV-2 [30].

In the quest of identifying COVID-19 waves which is a matter of utmost importance both for research and decision making, a study was carried out by Ayala *et al.* [31]. They used three different criteria to define the duration of a wave, and performed a sensitivity analysis using multivariate linear models to show their commonalities and differences. It was shown that defining a COVID-19 wave is not necessarily simple. The results highlight the need to adopt well-defined and well-justified definitions for COVID-19 waves [31].

This study seeks to build upon recent research on the impact of vaccination on COVID-19 dynamics, determining how long a current wave could last and investigating other imperative aspects to do with the disease. Here, we fit a SEIRV model to South African data for all the waves, determine feasible parameter values of the model and use these to make further numerical simulations, analysis and forecasts. Particularly, we aim to use the SEIRV model framework to determine the transmission dynamics of COVID-19. A detailed qualitative analysis is carried out to infer the long-term behaviour of the model solutions, thus depicting the long term epidemic trends. The model is calibrated using data from the different disease waves in South Africa. The fitted parameter values are used to investigate the role of vaccination (by quantifying the effect of vaccination rates and efficacy), and estimating how long a wave could last.

Thus, the remainder of this article is structured as follows. In §2, we present an SEIRV model assuming vaccination of susceptible, exposed and recovered populations. In §3, we explore the existence of an invariant region, determine the disease-free equilibrium, and derive the expression for the reproduction number $R_{0}$. Section 4 focuses on parameter identification for the model parameters based on South African data. Section 5 contains numerical investigations, simulations and a model forecast for the fifth wave to demonstrate the validity of our model. In §6, we perform global and local sensitivity analyses to determine the most important parameters influencing the dynamics of COVID-19 in South Africa. Finally, we conclude with a discussion in §7.

## 2. Model formulation

We propose a deterministic mathematical model to explore several scenarios to do with vaccination and other non-pharmaceutical control measures such as social distancing, government action and face mask usage (which we aggregate under one parameter *ϕ*). The model classifies the population into five classes at times *t*: the population of the susceptible individuals *S*, exposed individuals that are infected but not yet infectious *E*, vaccinated individuals *V*, infectious individuals *I* and recovered individuals *R*. Thus the total population is given by *N*(*t*) where *N* = *S* + *E* + *I* + *R* + *V*, where susceptible individuals are vaccinated at a rate *θ*. Here, we denote by Λ the recruitment rate of individuals into the population, *μ* the natural mortality rate, ε the vaccine efficacy, *κ* the average latent time and *δ* the disease-induced death rate. The infected individuals are treated at a rate *γ*. Given our force of infection, it is worth noting that, even though there is currently no cure against COVID-19, we assume that the vaccinated individuals have a reduced rate of becoming infected. Thus, vaccinated individuals are infected with COVID-19 at a rate (1−ε)λ with 0<ε<1 signifying the protective factor (efficacy) of the vaccine. We assume a force of infection with COVID- 19, *λ*, given by

**Figure 1.**
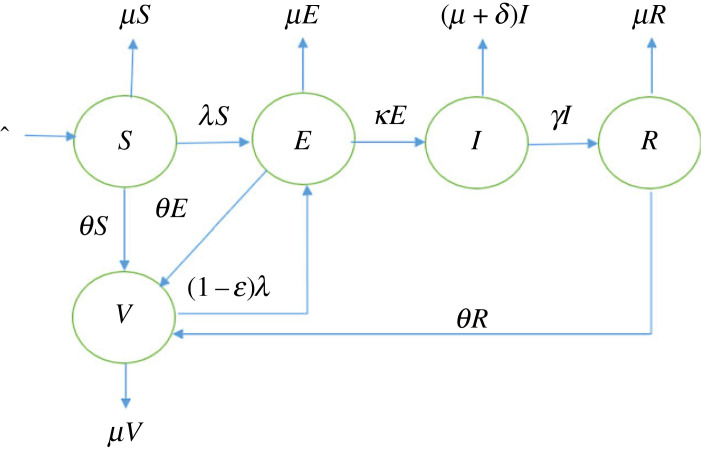
A schematic diagram of the model framework.

2.1 $$\lambda =\frac{\beta (1-\phi )I}{K+I^{q}},$$

where *β* is the probability of transmission by a COVID- 19 infectious individual and *q* is a scaling factor, which determines the degree of the Holing-type function (we take *q* ∈ {0, 1, 2, 3}). Initially, there was no vaccination and several countries only started vaccinating against COVID-19 after a year. Thus, the vaccination function that we consider is given by

$$\theta (t)=\left\{ \begin{array}{cc} 0,\; & 0\leq t\leq t_{1}\;\\ f\;(t),\; & t_{1}\leq t\leq t_{2},\; \end{array}\right.\;$$

where *t* is time, *t*_1_ the time when vaccination was started and *t*_2_ is the maximum modelling time. Figure 1 shows the schematic of the model and all the model parameters are stated in table 1.

**Table 1.** Model parameters and their interpretations.

parameter	description	units
Λ	recruitment of individuals in the susceptible population	number of individuals per day
*λ*	force of infection function	per day
*β*	transmission rate	per day
*ϕ*	COVID-19 control measures	dimensionless
*K*	50% of individuals being infected	half-saturation constant
*q*	scaling parameter	dimensionless
*μ*	natural mortality rate	per day
*θ*	vaccination function	per day
ε	vaccination efficacy	dimensionless
1/*κ*	average latent time	days
*γ*	recovery rate	per day
*δ*	disease-induced death rate	per day

A mathematical model, which can be derived from figure 1 is postulated as follows:

2.2*a* $$\frac{dS}{dt}=\Lambda -\lambda S-(\mu +\theta (t))S,$$

2.2*b* $$\frac{dE}{dt}=\lambda S+(1-\varepsilon )\lambda V-(\kappa +\theta (t)+\mu )E,$$

2.2*c* $$\frac{dI}{dt}=\kappa E-(\gamma +\mu +\delta )I,$$

2.2*d* $$\frac{dR}{dt}=\gamma I-(\theta (t)+\mu )R$$

2.2*e* $$\;\mathrm{and}\;\frac{dV}{dt}=\theta (t)(S+E+R)-[(1-\varepsilon )\lambda +\mu ]V,$$

together with non-negative initial conditions

$$S(0)=S_{0},\;E(0)=E_{0},\;I(0)=I_{0},\;R(0)=R_{0},\;V(0)=V_{0}.$$

## 3. Model analysis

### 3.1. Feasible region Ω

To guarantee that system (2.2) is mathematically well-posed in a feasible region Ω, we provide that

3.1 $$\Omega =\left\{(S(t),E(t),I(t),R(t),V(t))\in R_{+}^{5}\;:\;N(t)\leq \frac{\Lambda }{\mu }\right\}.$$

**Theorem 3.1.**

*There exists a domain* Ω, *shown in equation* (*3.1*), *in which the solution set* {(*S*(*t*), *E*(*t*), *I*(*t*), *R*(*t*), *V*(*t*))} *with the given non-negative initial conditions is contained and bounded for all* *t* ≥ 0.

For the proof of theorem 3.1, refer to appendix A.

### 3.2. Disease-free state

The disease-free equilibrium (DFE) point describes the absence of COVID-19 in the population, and the endemic equilibrium (EE) point which exists at any positive prevalence of COVID-19 in the population. Due to the recruitment terms Λ, there are no trivial equilibrium points. This implies that (S*, E*, I*, R*, V*) ≠ (0, 0, 0, 0, 0, 0, 0). We set all the infectious compartment to zero (0) at the DFE point *E*_0_: E* = 0, I* = 0, R* = 0, and (S*, E*, I*, R*, V*) = (S*, 0, 0, 0, V*) for the system (2.2). Solving the right-hand sides of equations (2.2*a*) and (2.2*e*) of the system (2.2) equated to zero, yields

$$S^{*}=\frac{\Lambda }{\mu +\theta }\;\text{and}\;V^{*}=\frac{\theta \Lambda }{\mu (\mu +\theta )}.$$

Thus, we obtain

$$E_{0}=\left(\frac{\Lambda }{\mu +\theta },\;0,\;0,\;0,\;\frac{\theta \Lambda }{\mu (\mu +\theta )}\right).$$

### 3.3. Basic reproduction number

Epidemiological models usually have a threshold parameter, called the basic reproduction number, $R_{0}$. This threshold parameter enables us to determine the transmission and spread of the disease in the study population, such that if $R_{0}<1$, then the DFE is locally asymptotically stable, implying that the population cannot be invaded by the disease, but if $R_{0}>1$, then the DFE is unstable and invasion is certain. $R_{0}$ is defined as the average number of secondary cases produced by a ‘typical’ infected (assumed infectious) individual during his/her entire life as infectious when introduced in a population of susceptible. We employed the next generation matrix technique [32] to obtain $R_{0}$, given by

3.2 $$R_{0}=\frac{\beta (1-\phi )\;\Lambda \;\kappa \;[\mu +(1-\varepsilon )\;\theta ]}{K\;\mu \;(\mu +\theta )\;(\kappa +\theta +\mu )\;(\gamma +\mu +\delta )}.$$

From the inspection of equation (3.2), the calculated basic reproduction number shows that NPIs and vaccination reduce the disease incidence. Particularly, the effect of an increase in *θ* on *R*_0_ is greater for time interval [*t*_1_, *t*_2_].

The local stability of the DFE point is obtained from the basic reproduction number $R_{0}$, and can be summarized as follows.

**Theorem 3.2.**

*If* $R_{0}<1$ *the disease-free state* *E*_0_ *is locally asymptotically stable. Otherwise, it is unstable if* $R_{0}>1$.

The proof of theorem 3.2 can be found in appendix A. The global stability analysis of system (2.2) can be inferred by constructing a suitable Lyapunov function. Refer to appendix A for details. In addition, the bifurcation and the existence of EE states and their number can be found in appendix A.

Having looked at the mathematical analysis of system (2.2), we next consider the problem of parameter identification of the model using South African COVID-19 data.

## 4. Model fitting and parameter estimations

Unlike other studies that did not calibrate their models or considered only a single wave [7,16,17], the present model is individually parameterized and fitted to the four different waves. Estimates for other model parameters and their ranges were obtained from the literature [33]. Parameter values are estimated using the cumulative recovery data from South Africa, obtained from the github repository [34]. To take into account under reporting, daily infection numbers were multiplied by a detection probability, *p*, which is also estimated. The rest of the unknown parameters were estimated by formulating a least-squares algorithm, with an aim of minimizing the distance between daily recorded cumulative recovered cases and the model output. The objective function to be minimized is the root mean square error defined by

4.1 $$f_{j}=\sqrt{\frac{\sum_{i=1}^{N}{\left(A_{i}^{c}-A_{i}^{d}\right)}^{2}}{N}},$$

where *A*^*d*^ represents the vector of daily recorded cumulative cases of recovered individuals within the period of consideration of length *N* while *A*^*c*^ is the corresponding model output.

**Figure 2.**
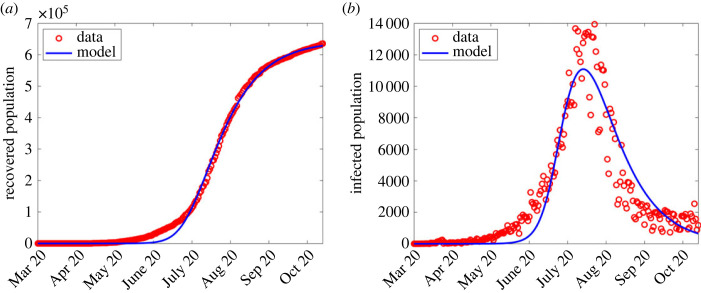
Simulation output results using fitted parameter values in the first wave shown in table 2. (*a*) Simulation output plotted alongside cumulative recoveries. (*b*) Simulation output plotted alongside daily reported cases.

**Figure 3.**
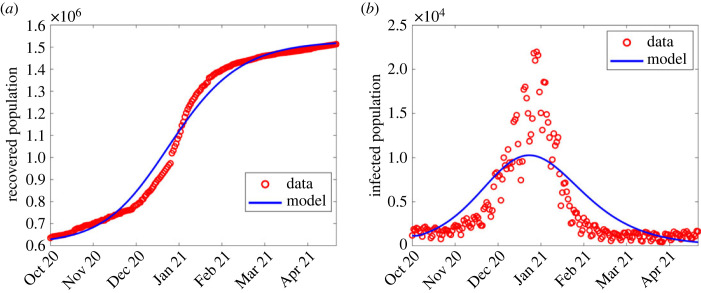
Simulation output results using fitted parameter values in the second wave shown in table 2. (*a*) Simulation output plotted alongside cumulative recoveries. (*b*) Simulation output plotted alongside daily reported cases.

**Figure 4.**
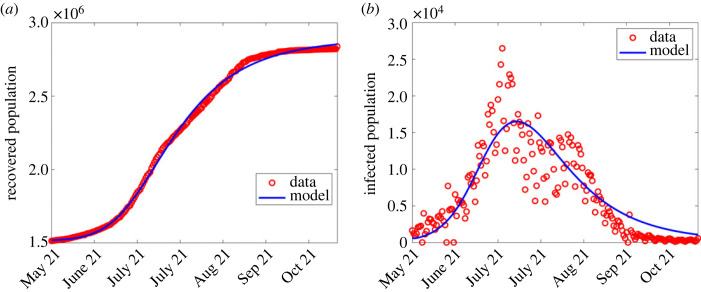
Simulation output results using fitted parameter values in the third wave shown in table 2. (*a*) Simulation output plotted alongside cumulative recoveries. (*b*) Simulation output plotted alongside daily reported cases.

**Figure 5.**
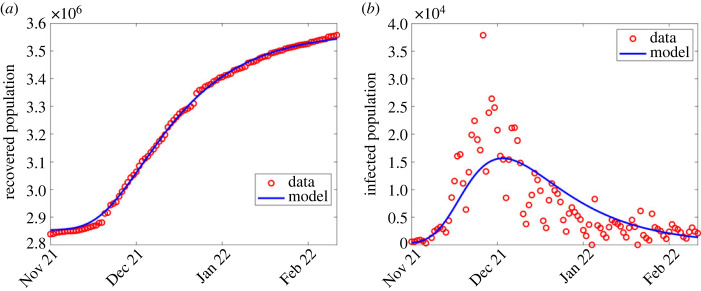
Simulation output results using fitted parameter values in the fourth wave shown in table 2. (*a*) Simulation output plotted alongside cumulative recoveries. (*b*) Simulation output plotted alongside daily reported cases.

**Figure 6.**
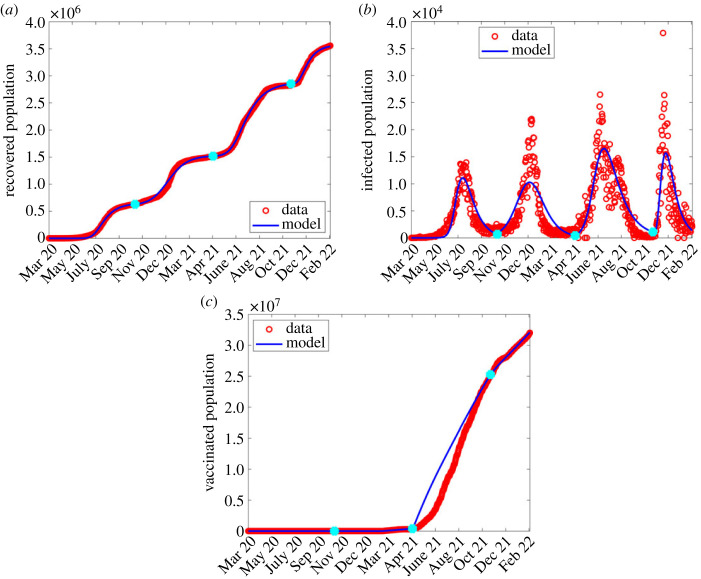
Combined waves with parameter values listed in table 2 and *λ* from equation (2.1), where blue dots are wave joints. (*a*) Cumulative recovery; (*b*) daily infection numbers; (*c*) vaccinated population.

The least-squares minimization problem in Matlab is solved using the ‘fminsearchbnd’ function [35,36]. This function allows parameter estimation from a bounded parameter range. We then identified points at which each wave began, and hence implemented the parameter estimation algorithm from the start of each wave to the end of that wave period, which is also the beginning of a subsequent wave. The estimated parameter values are shown in table 2. The results are shown in figures 2–6. In the first wave, we fitted all parameters, except Λ and *μ* which were fixed according to [37]. Since from equation (2.1) the term *β*(1 − *ϕ*) can be aggregated into a single parameter, we fixed *ϕ* = 0.5 for all the waves and only estimated *β*, while *θ* = 0 and ε are fixed in the first wave since vaccination had not started. See figure 2 for the comparison of simulation output using estimated parameter values and the observed numbers during the first wave.

**Table 2.** Table of fitted parameters for all the waves with the force of infection, *λ*, given in equation (2.1).

parameters	parameter values			
	wave 1	wave 2	wave 3	wave 4
Λ	3468	fixed	fixed	fixed
*β*	0.0969	0.4320	0.0958	0.1187
*ϕ*	0.5	fixed	fixed	fixed
*K*	6.1186 × 10^6^	6.0035 × 10^7^	9.4589 × 10^6^	3.6715 × 10^6^
*q*	1.0012	1.0000	1.0015	1.0091
*μ*	4.4159 × 10^−5^	fixed	fixed	fixed
*θ*	0	0.0001	0.0027	0.0013
ε	0	0.5068	0.5072	0.8079
1/*κ*	4.9552 (fitted)	fixed	fixed	fixed
1/*γ*	8.3018 (fitted)	fixed	fixed	fixed
*δ*	0.0078 (fitted)	fixed	fixed	fixed
detection rate	0.0133	0.0240	0.0346	0.0138

In the second wave, most of the parameters were fixed from the first wave, and only estimated *β*, *K*, *q*, *θ*, ε and the detection probability, *p*. See the third column of table 2 for estimated parameters corresponding to the second wave and figure 3 for the comparison between the model output and observed numbers.

The estimation of parameters in the third and fourth waves followed a similar manner as in the second wave. The estimated parameter values are shown in the fourth and fifth columns of table 2 and comparison of results shown in figures 4 and 5, respectively.

Following the estimation of parameters in all the four waves, we concatenated time series of simulated values for all the four waves and plotted them alongside cumulative recovered population, daily reported cases and cumulative vaccinated population as shown in figure 6.

Next, we present simulation results on the effect of varying the contact rate *β*, the intervention rate *ϕ*, vaccination rate *θ* and the vaccine efficacy ε on infectious population. We use the definition of *λ* from equation (2.1).

## 5. Numerical simulations

In general, data for COVID-19 waves 1–4 were explored first to observe any changes for *λ* defined in (2.1), and compare the observed infections to the model output. The computed infections agree well with the observed infections as shown in figures 2–5.

Except for the first and second waves (in figures 2 and 3), the profiles of disease dynamics in the third and fourth waves (figures 4 and 5) follow similar trends. However, what is surprising is the profile of infected individuals which displays a sharp rise at the beginning of the third and fourth waves before it decreases. The results are consistent with a study of Madhi *et al.* [38] who reported that incidence of SARS-CoV-2 infection increased and subsequently declined more rapidly during the fourth wave than it had during the three previous waves in Gauteng province, South Africa. Probably the observed trend may be attributed to the discovery of the new and potentially more transmissible variant of novel-corona virus B.1.1.529 named ‘Omicron’ which heavily affected the population of South Africa from mid-2020 to December 2021 [39,40]. Moreover, the vaccinated population remained low [41], thereby accelerating the transmission. In addition, even during the presence of vaccine, Madhi *et al.* [38] reported that vaccinated participants were more likely to be seropositive for SARS-CoV-2 than unvaccinated participants.

### 5.1. Effect of varying interventions on daily infections

To investigate the effects of intervention measures on daily infections, we simulated system (2.2) for all four waves, with varying control parameters: non-pharmaceutical interventions (*ϕ*) and pharmaceutical interventions (*θ* and ε).

#### 5.1.1. Effect of varying non-pharmaceutical interventions

**Figure 7.**
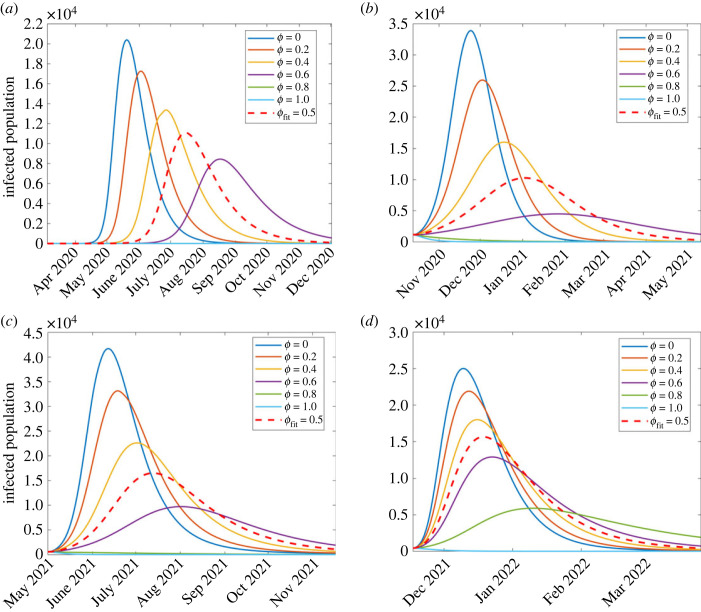
Simulation results of model (2.2) showing the effect of NPIs (*ϕ*) on the infected population for the four waves of the South Africa COVID-19 epidemic: (*a*) first wave, (*b*) second wave, (*c*) third wave and (*d*) fourth wave.

The effect of varying non-pharmaceutical control measures modelled by *ϕ* on the disease dynamics is investigated for all four waves. The results are shown in figure 7. It can be seen from all the four waves that as *ϕ* increases, the peak infection decreases, signifying the impact of non-pharmaceutical interventions (NPIs) such as social distancing and stay-indoor scenarios on incidence rates, consistent with [42–44]. From figure 7, it is evident that NPIs were more effective in the first and second waves than in the third and fourth, as attributed to the fact that South Africa had started vaccination in the third and fourth waves.

In the first wave, shown in figure 7*a*, the red dashed curve shows the disease dynamics with the estimated parameter values in table 2 under the first wave column. With these parameter values and *ϕ* = 0.5, the highest number of infections estimated are 11 094 people, on 15 July 2020. However, as *ϕ* is increased to 0.6, peak infections decrease to 8450 on 17 August 2020. This shows that an increase in an intervention may decrease the number of infections to a level that hospitals and government could handle; however, the disease would last longer. Furthermore, simulating a scenario where we assume government implements stringent measures, *ϕ* ≥ 0.8, an epidemic does not start and the disease dies out. This result agrees with Stokes *et al.* [44] whose study found that strict measures such as early school and workplace closures were associated with lower COVID-19 mortality rates. If the interventions are reduced, that is, a step-wise decrease in *ϕ* from 0.5, 0.4, 0.2 and 0, the infection peaks increase, respectively, to 13 369 (27 June), 17 253 (2 June) and 20 398 (27 June 2020) implying that the first wave would have ended earlier, before July 2020.

Similar observations are made with the remaining three waves. In the second wave, shown in figure 7*b*, the estimated highest number of infections (corresponding to the red dashed arrow) is 10 273 (3 January 2021), simulated with parameter values in table 2. As *ϕ* varies from 0, 0.2, 0.4 and 0.6 then peak infections change, respectively, from 33 914 (24 November 2020), 25 952 (3 December 2020), 16 028 (19 December 2020) to 4495 (26 January 2021). For *ϕ* value after 0.8, an epidemic does not occur and hence no wave is formed, perhaps signifying possible outcomes of implementing stronger NPIs [45].

For the third wave (figure 7*c*), the estimated peak infections (corresponding to the red dashed arrow) were 16 489 (13 July 2021), simulated with parameter values in table 2, under wave three column. As the value of *ϕ* changes from 0, 0.2, 0.4 and 0.6 then peak infections change, respectively, from 41 703 (12 June 2021), 33 143 (12 June 2021), 22 612 (2 July 2021) to 9709 (2 August 2021). Similarly to the previous waves, there is no epidemic for *ϕ* ≥ 0.8.

Lastly, regarding the fourth wave (figure 7*d*), the estimated peak infections (corresponding to the red dashed arrow) were 15 643 (19 December 2021), simulated with parameter values in table 2, under wave four column. As we investigate the effect of varying NPIs, we increased values of *ϕ* from 0, 0.2, 0.4 0.6 and 0.8 which resulted in the respective peak infections changing from 25 018 (10 December 2021), 21 906 (12 December 2021), 18 004 (16 December 2021), 12 923 (23 December 2021) to 5898 (10 January 2022). In all four waves, varying *ϕ* results in similar qualitative dynamics. Here, we also varied *ϕ* while keeping all the remaining parameters fixed. To investigate the optimal combination of pharmaceutical and NPIs sufficient to control COVID-19, we look at the heat maps of $R_{0}$ with pairwise variations of pharmaceutical and non-pharmaceutical parameter values (figure 14).

#### 5.1.2. Effect of varying pharmaceutical interventions

**Figure 8.**
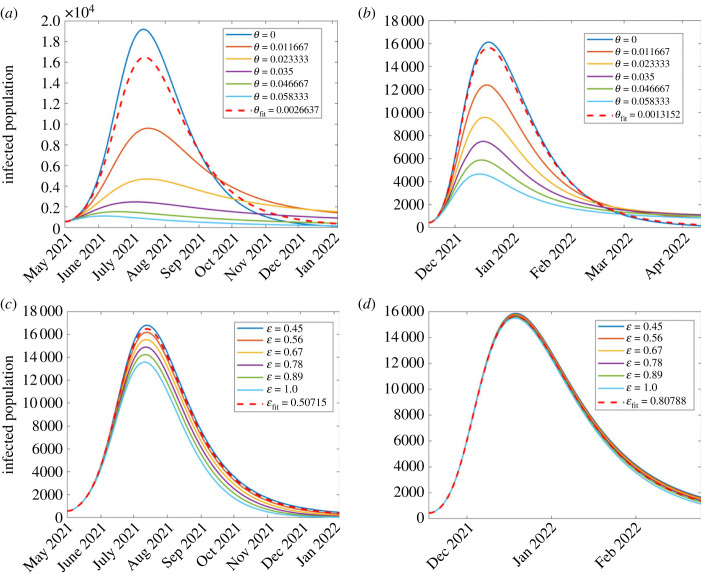
Effect of varying parameter values of *θ* and ε which, respectively, represent vaccination rate and vaccination efficacy, on the disease dynamics among infectious population. (*a*) First wave; (*b*) second wave; (*c*) third wave; (*d*) fourth wave. The figure depicts a reduction in disease incidence with increasing vaccination rates. Both parameters were more influential in the third wave than in the fourth.

In this section, we investigate the effect of pharmaceutical interventions. That is, the effect of vaccinations. We therefore investigate the effect of vaccination rate *θ* and the vaccine efficacy, modelled as ε. Since COVID-19 vaccination was initiated towards the end of the second wave, our investigation here focused on the third and fourth waves. The results are displayed in figure 8. In all the graphs, the red dashed curve shows simulation with the corresponding fitted parameters in each wave.

*Effect of vaccination rate, *θ*.* We investigate the effect of varying vaccination rate, *θ*. Since vaccination began towards the end of the second wave, the investigation was only done for two waves, namely the third and fourth. The results are displayed in figure 8*a*,*b*. It was observed, from figure 8*a*,*b*, that higher vaccination rates reduce the disease incidence. These results are consistent with Watson *et al.* [24] who reported that more COVID-19 related deaths could be averted if vaccine coverage (rate) is increased. Although it has been widely reported that vaccinated populations experience COVID-19 fatality rates lower than non-vaccinated [46,47], figure 8 depicts that the reverse is true. Perhaps, the results might raise an important question: was the vaccine used effectively enough? leading us to the subject of interest in §5.1.2. We observe that in general, vaccination reduces the number of infections. In the third wave, results shown in figure 8*a*, the highest infection rates occur when *θ* = 0. This implies that if there were no vaccination, then the peak infections would have occurred on 12 July 2021 with daily infections being 19 193. As *θ* increases taking the values 0.011, 0.02 and 0.035, then peak infection decreases, respectively, to 9617 (16 July 2021), 4698 (16 July 2021) and 2447 (4 July 2021). For *θ* = 0.04 and 0.05, then the infections start and die out almost immediately. Simulation with the fitted vaccination parameter *θ* = 0.00266 gives peak infection of 16 498 and occurred on 13 July 2021.

In the fourth wave, figure 8*b*, we see similar qualitative dynamics to the third wave. Simulation with the estimated vaccination rate *θ* = 0.00132 gives rise to peak infection of 15 643 and occurred on 19 December 2021. An increase in vaccination rate from the estimated vaccination resulted in decreased infection rates, with the infection peaks decreasing from 12 405 (18 December 2021), 9584 (17 December 2021), 7492 (16 December 2021), 5874 (15 December 2021) and 4557 (14 December 2021). With no vaccination, then peak infection would have been 16 128.

*Effect of vaccination efficacy, ε.* Recently, di Lego *et al*. [48] argued that unless vaccinated people are also tested for COVID-19 infection, it is difficult to unravel the effect of vaccines in reducing case fatality rate (CFR). Therefore, the effect of varying vaccine efficacy rate ε on the dynamics of the disease among the infectious population was investigated for the second, third and fourth waves. The results are displayed in figure 8*c*,*d*. The results in figure 8*c*,*d* show that a higher vaccination rate reduces the disease incidence although by smaller margins in the fourth wave. Moreover, for most countries, studies have shown that despite a high proportion of vaccinated individuals, the CFR does not significantly decrease for almost a year after vaccine uptake [48–51].

### 5.2. Model forecasting

**Figure 9.**
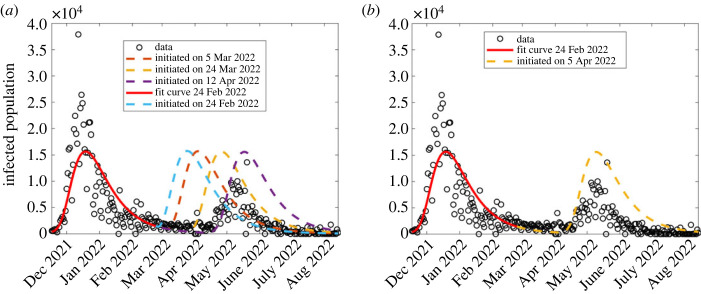
Model forecast of the COVID-19 dynamics to generate the fifth wave, varying the date at which the wave is initiated. The solid red curve shows model simulation to 24 February 2022, the end of region considered for the model fitting, while the dashed curves indicate forecasted dynamics.

**Figure 10.**
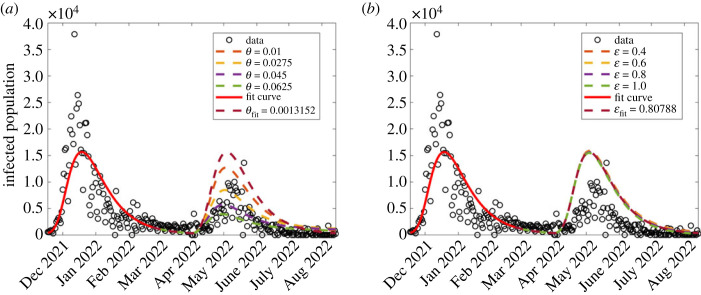
Investigating the effect of varying vaccination rate *θ* and vaccine efficacy ε on the forecasted COVID-19 dynamics corresponding to model (2.2). (*a*) Varying vaccination rate, *θ*; (*b*) varying vaccine efficacy, ε.

**Figure 11.**
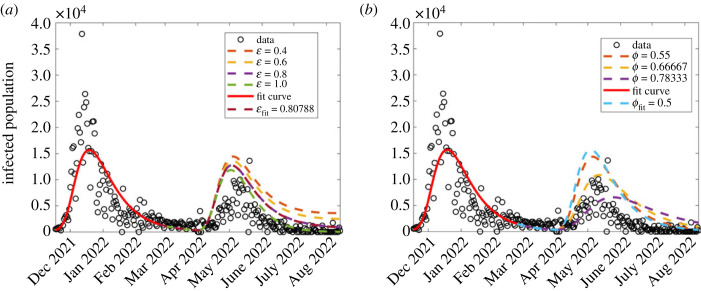
The combined effect of varying vaccination rate, *θ*, and vaccine efficacy, ε, on the forecasted results of model (2.2). (*a*) Vaccination rate is fixed to *θ* = 0.01 while varying ε; (*b*) varying *ϕ*.

In this section, we forecast the model output to generate the predicted fifth wave, using the approximated model parameters in the fourth wave (the wave four column of table 2), and compare this to the South African COVID-19 data corresponding to the fifth wave. See results in figures 9–11.

First, we sought to forecast the model output, considering different dates for initiating the fifth wave. Hence, we generate different scenarios, each corresponding to a different date of initiating the wave as shown in figure 9, with parameter values generated from the previous (fourth) wave. From this, we identified the closest scenario which corresponds to a wave initiated on 5 April 2022 as shown in figure 9*b*. From this scenario, the forecasted model output predicts higher infection rates than the observed; therefore we sought to analyse the dynamics of this scenario, by changing the control parameters (*θ*, ε and *ϕ*), to identify parameter values which generate forecasted results as close as possible to the observed data.

Having obtained a date of initiating a wave that describes as close as possible the observed daily infection numbers, next, we proceeded to investigate the effect of varying vaccination parameters *θ* and ε on the model forecast dynamics as shown in figure 10. While fixing all the other parameters and varying only the vaccination rate, *θ*, our forecasted fifth wave results (figure 10*a*) described the COVID-19 observed infection numbers for some parameter values (higher vaccination rate than in fourth wave). Consistent with Watson *et al.* [24], our model forecasts mean that there was an increased vaccination, or possibly increased immunity due to larger population’s exposure to the disease, and hence low observed daily infection numbers in the fifth wave.

When fixing vaccination rate low (*θ* = 0.0013), vaccine efficacy seems to not have contributed significantly to the reduced daily infection numbers in the fifth wave (figure 10*b*); however, we note from figure 11*a* that when vaccination rate is increased slightly to about *θ* = 0.01, then ε has a significant contribution to a reduction in daily infection numbers. This implies that COVID-19 observed dynamics are due to different intervention strategy combinations. We also sought to forecast COVID-19 dynamics with changing non-pharmaceutical interventions (*ϕ*) as shown in figure 11*b*. We observe that increasing *ϕ* leads to a decrease in the daily infection numbers, as observed in the data.

## 6. Sensitivity analysis

In modelling infectious diseases, care should be taken on the input parameters, since their uncertainty determines the disease dynamics and the extent of progression. To overcome this challenge, sensitivity analysis is employed to help measure the adequacy and robustness of the model and to determine which parameters substantially affect the model output [52–55]. In this study, we considered both local and global sensitivity analysis to investigate the influence of model parameters θ, β, κ, K, ε, γ, ϕ and δ on the basic reproduction number $R_{0}$. We did not investigate the sensitivity with respect to the parameters Λ and μ since they were also kept fixed throughout the fitting process and simulations.

### 6.1. Local sensitivity analysis

The direct differential method (DDM) is used for carrying out local sensitivity analysis. The DDM allows us to fully characterize each sensitivity index as a function of the independent variable [56,57]. Therefore, given the analytic expression of the reproduction number, we differentiate it with respect to each parameter and evaluate the resulting derivatives with the estimated (nominal) parameters for each wave. The local sensitivity analysis is described as, given $R_{0}$ and a parameter, say *p*, then the sensitivity of $R_{0}$ with respect to *p*, denoted $S_{p}^{R_{0}}$, is given by

6.1 $$S_{p}^{R_{0}}=\frac{\partial R_{0}}{\partial p}.$$

Due to the differences in magnitudes of the parameters, equation (6.1) is modified by computing the sensitivity ${ST}_{p}^{R_{0}}$ of the logarithm of values as

6.2 $${ST}_{p}^{R_{0}}=\frac{\partial \log(R_{0})}{\partial \log(p)}=\frac{\;p}{R_{0}}\cdot S_{p}^{R_{0}}.$$

**Figure 12.**
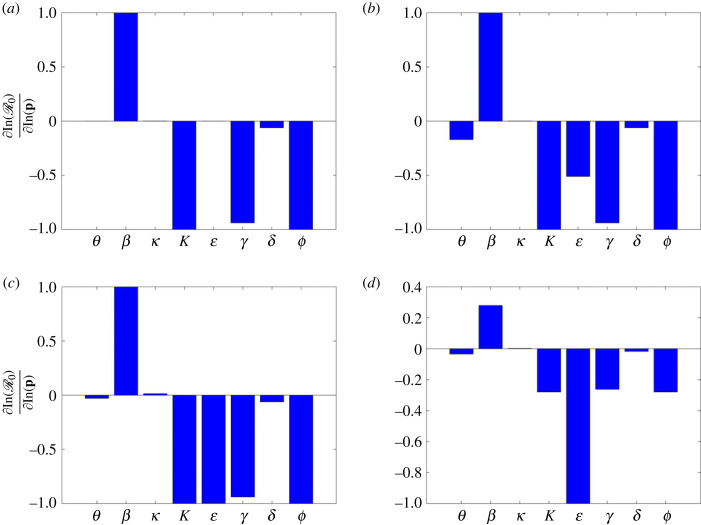
Local sensitivity analysis results of the basic reproduction number $R_{0}$ (3.2) corresponding to (*a*) first wave, (*b*) second wave, (*c*) third wave and (*d*) fourth wave. For each wave, the parameters from the respective columns of table 2 were used as nominal parameter values.

Normalized sensitivity indices were computed independently for each wave with the respective nominal parameter values given in table 2. Local sensitivity analysis results are shown in figure 12. The sensitivity values are scaled to lie in the interval [− 1, 1]. It is clearly seen from the figure that the local effect of parameters is dependent on the base parameter value. From the global sensitivity analysis, the most influential parameters on the disease dynamics are the contact rate, NPIs, vaccination rate and vaccine efficacy. Further, infinitesimal changes in vaccine efficacy greatly influence the disease progression. A tiny increase in the vaccine efficacy leads to a bigger decrease in the basic reproduction number *R*_0_. As expected, the vaccine efficacy is mostly influential in the third and fourth waves.

In the first wave, as shown in figure 12*a*, the dominant parameters are *β*, *K*, *γ* and *ϕ*; the transmission rate *β* has a positive effect on $R_{0}$, which is in contrast to the other parameters. COVID-19 vaccinations had not been initiated during the first wave, hence *θ* = 0 and ε=0. In the remaining waves, the dominant parameter is ε whose increase leads to a decrease in $R_{0}$. Therefore, we can conclude that infinitesimal changes in vaccine efficacy greatly influence the disease progression.

### 6.2. Global sensitivity analysis

The partial rank correlation coefficient (PRCC) [58,59] is used to carry out the global sensitivity analysis. The Latin hypercube sampling (LHS) was used to independently obtain 10 000 samples from each parameter and hence 10 000 runs were made per simulation. Since the distribution of the parameters is unknown, we used uniform distribution for each parameter, identifying maximum and minimum such that all the realistically possible values are considered, covering the entire range of estimated parameters for each wave as shown in table 2. Table 3 contains ranges of parameter values used to carry out global sensitivity analysis. In addition, the table also contains the PRCC and *p*-values for each parameter.

**Table 3.** Range of parameters used for sensitivity, together with their PRCC values and the corresponding *p*-values.

parameters	*θ*	*β*	*κ*	*K*	ε	*γ*	*δ*	*ϕ*
range	[1 × 10^−16^, 1]	[1 × 10^−16^, 1]	[1/8, 1/2]	[1 × 10^3^, 600419996]	[1 × 10^−16^, 1]	[1/18, 1/3]	[1 × 10^−16^, 1/6]	[1 × 10^−16^, 1]
PRCC	−0.4222	0.6939	0.2552	−0.6941	−0.6941	−0.3405	−0.2296	−0.6949
*p*-value	0	0	8.0899 × 10^−295^	0	0	0	2.2060 × 10^−211^	0

**Figure 13.**
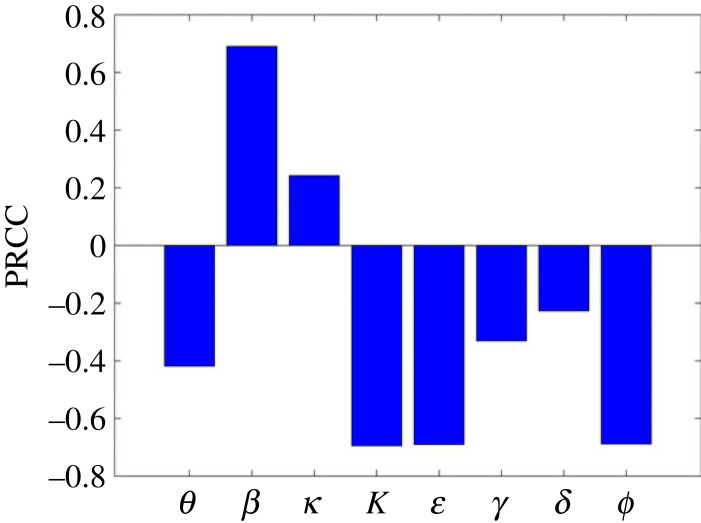
Partial rank correlation coefficient (PRCC) values showing the sensitivity of the basic reproduction number $R_{0}$ (3.2) with respect to the input parameters.

Figure 13 shows the global sensitivity analysis results. From the figure, *β* and *κ* are the only parameters which have significant positive PRCC values. However, the PRCC value for *κ* is approximately 0.39 implying that this parameter does not significantly affect $R_{0}$; on the other hand increasing *β* increases $R_{0}$. Physically this means that increasing the transmission rate increases the spread of the disease. By contrast, the parameters θ, K, ε ϕ, δ and *γ* have negative PRCC values, implying that an increase in these parameters leads to a reduction in $R_{0}$. Comparing local and global sensitivity analysis, it can be seen that the effect of parameters on $R_{0}$ is qualitatively similar.

**Figure 14.**
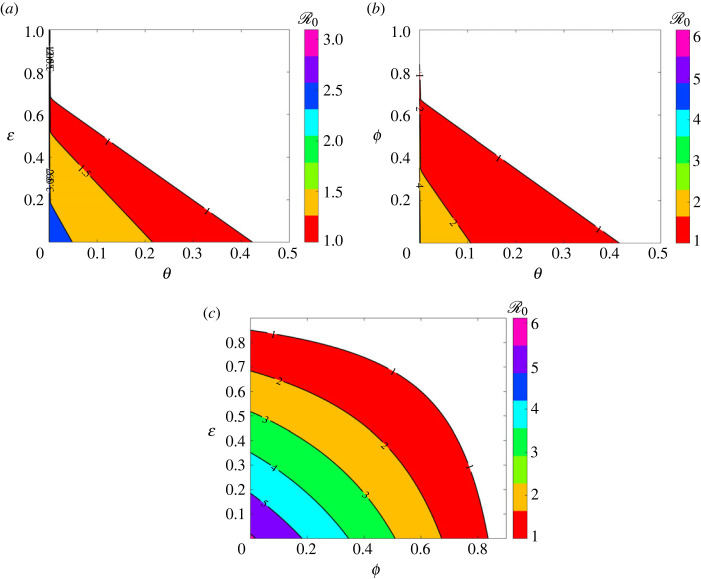
Heat map of $R_{0}$ as a function of control parameters, where $R_{0}<1$ in the colourless region. (*a*) $R_{0}$ as a function of *θ* and ε, (*b*) $R_{0}$ as a function of *θ* and *ϕ* and (*c*) $R_{0}$ as a function of *ϕ* and ε. The remaining parameters are fixed as shown in table 2.

To further supplement the sensitivity results, contour plots of $R_{0}$ for different parameter combinations (while keeping others fixed) were plotted. Figure 14 shows heat maps of the reproduction number as a function of pairwise control parameters, the vaccination rate *θ*, the vaccine efficacy ε and the control parameter *ϕ*. Figure 14*a* depicts that the epidemic would better be controlled with a bigger vaccination efficacy than the vaccination rate in order to have $R_{0}<1$. For example, a 70% vaccine efficacy would only require a vaccination rate of *θ* = 0.1. Figure 14*b* shows that the vaccination rate is more influential than NPIs and thus a bigger vaccination rate with considerable usage of the other measure would guarantee $R_{0}<1$. For example, a vaccination rate of *θ* = 0.4 would only require *ϕ* = 0.1 to ensure that $R_{0}<1$. Figure 14*a* depicts that a combination of vaccination efficacy and NPIs would only yield optimal results if they are all used in large quantities.

## 7. Conclusion

In this paper, an SEIRV model framework has been presented for analysing the transmission dynamics of COVID-19. The main aim of this article was to investigate the effect of COVID-19 vaccination on possible duration of wave occurrences and to determine the effect of vaccination rate and efficacy. A thorough qualitative analysis was carried out. This included determining model equilibria and their stability, determining *R*_0_, investigating the existence of backward bifurcation and determining the existence of an EE. The model was calibrated using South African reported data for COVID-19 from the start of the epidemic until February 2022 and data for the fifth wave was used to test the model predictability. Local and global sensitivity analyses were carried out in order to determine the factors, in the form of parameters, that most influence disease progression via the basic reproduction number.

This study found that the combination of NPIs with vaccination would certainly beat COVID-19 even in the absence of a cure for the disease. It has further been observed that massive vaccination would only be beneficial if a highly effective vaccine is used. Moreover, further studies could consider constructing a force of infection that replicates all waves rather than fitting the model to individual waves. Depending on data availability, it could as well be interesting to consider an age-based model which would provide deeper insights on the disease dynamics in different age groups. Further studies could investigate how the disease incidence changes if vaccine induced immunity wanes or if there are vaccine resistant sub-types.

## Data Availability

All the data used can be accessed within the paper, and simulations produced via the described algorithms. Appropriate citations and links have also been provided. In addition to that, South African COVID-19 data were accessed through the public github link at https://github.com/owid/covid-19-data/tree/master/public/data. The global sensitivity analysis was performed with the publicly available code at http://malthus.micro.med.umich.edu/lab/usadata/. The rest of the codes have been deposited to Dyrad. The authors confirm that the data supporting the findings of this study are available within the article and via public repository [60].

## Appendix A. Mathematical analysis

***Proof of theorem 3.1.***
We obtain the total dynamics of the population by adding the equations of system (2.2). This yields

N(t)=S(t)+E(t)+I(t)+R(t)+V(t).

It then follows that

$$\frac{dN}{dt}=\Lambda -\mu N-\delta I\leq \Lambda -\mu N,$$

so that

A 1 $$\frac{dN}{dt}+\mu N\leq \Lambda .$$

The solution to the first-order linear differential inequality (A 1) is given as

A 2 $$N(t)\leq \frac{\Lambda }{\mu }(1-\exp(-\mu t)\;)+N(0)\exp(-\mu t).$$

As t⟶∞, we have N(t)≤Λ/μ. Therefore, *N*(*t*) is a bounded function of time. This implies that Ω is bounded and at limiting equilibrium $\lim_{t⟶\infty }N(t)=\Lambda /\mu$. System (2.2) is well-posed with all solutions in Ω remaining in Ω or in a neighbourhood of Ω if initial conditions are positive. Thus, Ω is positively invariant and is attracting with respect to the flow of System (2.2). ▪

**A.1. Basic reproduction number**

To calculate the basic reproduction number $R_{0}$, we employ the next generation matrix technique [32]. First, the infectious compartment model is expressed in the form

$$\frac{dx_{i}}{dt}=F_{i}(x)-V_{i}(x),$$

where $F_{i}$ are the new infections and $V_{i}$ are the transfers of infections from one compartment to another. $V_{i}(x)=V_{i}^{-}(x)-V_{i}^{+}(x)$, $x\in R_{+}^{5}$, and *i* = 1, 2, …, *m* is the number of infectious compartments. It is assumed that each function is continuously differentiable at least twice in each variable. Evaluating at the DFE *E*_0_, we then have $F=[(\partial F_{i}/\partial x_{\;j})\;(E_{0})]$ and $V=[(\partial V_{i}/\partial x_{\;j})\;(E_{0})]$ with *i* ≤ 1 and *j* ≤ *m* such that $R_{0}$ is the maximum or dominant eigenvalue of the matrix formed from *FV*^−1^. In the case of system (2.2), we have

$$F=\left[\begin{array}{c} \lambda S+(1-\varepsilon )\lambda V\\ 0 \end{array}\right]\;\text{and}\;V=\left[\begin{array}{c} (\kappa +\theta (t)+\mu )E\\ \;(\gamma +\mu +\delta )I-\kappa E \end{array}\right].$$

Taking the derivatives of F and V at the DFE point *E*_0_, we have *F* and *V*, respectively,

$$F=\left[\begin{array}{cc} 0 & \frac{\beta (1-\phi )\;[S_{0}+(1-\varepsilon )\;V_{0}]}{K}\\ 0 & 0 \end{array}\right]$$

and

$$V=\left[\begin{array}{cc} \left(\kappa +\theta +\mu \right) & 0\\ -\kappa & \left(\gamma +\mu +\delta \right) \end{array}\right].$$

Hence, it follows that

$$V^{-1}=\left[\begin{array}{cc} {\left(\kappa +\theta +\mu \right)}^{-1} & 0\\ \frac{\kappa }{\left(\kappa +\theta +\mu )\;(\gamma +\mu +\delta \right)} & {\left(\gamma +\mu +\delta \right)}^{-1} \end{array}\right],$$

so that

$$FV^{-1}=\left[\begin{array}{cc} \frac{\beta (1-\phi )\;[S_{0}+(1-\varepsilon )\;V_{0}]\kappa }{K\;(\kappa +\theta +\mu )\;(\gamma +\mu +\delta )} & \frac{\beta (1-\phi )\;[S_{0}+(1-\varepsilon )\;V_{0}]}{K\;(\gamma +\mu +\delta )}\\ 0 & 0 \end{array}\right].$$

The basic reproduction number is the spectral radius of the product *FV*^−1^, that is, $R_{0}=\rho (FV^{-1})$. Hence, for system (2.2), we obtain

A 3 $$R_{0}=\frac{\beta (1-\phi )\;[S_{0}+(1-\varepsilon )\;V_{0}]\kappa }{K\;(\kappa +\theta +\mu )\;(\gamma +\mu +\delta )}.$$

Now, substituting the values of *S*_0_ and *V*_0_ into equation (A 3), yields

A 4 $$R_{0}=\frac{\beta (1-\phi )\;\Lambda \;\kappa \;[\mu +(1-\varepsilon )\;\theta ]}{K\;\mu \;(\mu +\theta )\;(\kappa +\theta +\mu )\;(\gamma +\mu +\delta )}.$$

Equation (A 4) is the expression of the reproduction number $R_{0}$ corresponding to system (2.2).

**A.2. Local stability of disease-free equilibrium point**

***Proof of theorem 3.2.***
We need to show that the Jacobian matrix, $J(E_{0})$, of system (2.2) has negative eigenvalues at the DFE, *E*_0_. Evaluating $J(E_{0})$ at *E*_0_, yields

A 5 $$J(E_{0})=\left[\begin{array}{ccccc} -(\mu +\theta ) & 0 & -\frac{\beta \;(1-\phi )\;S_{0}}{k} & 0 & 0\\ 0 & -(k+\theta ) & \frac{\beta \;(1-\phi )\;[S_{0}+(1-\varepsilon )]V_{0})}{K} & 0 & 0\\ 0 & k & -(\gamma +\mu +\delta ) & 0 & 0\\ 0 & 0 & \gamma & -(\mu +\theta ) & 0\\ \theta & \theta & \frac{-\beta \;(1-\phi )\;(1-\varepsilon )V_{0}}{K} & \theta & -\mu \end{array}\right].$$

The first and fifth columns of the Jacobian matrix in (A 5) contain only the diagonal terms which form the two negative eigenvalues, −(*μ* + *θ*) twice, and −*μ*. The remaining two eigenvalues are obtained from the sub-matrix

A 6 $$J_{2}(E_{0})=\left[\begin{array}{cc} -(\kappa +\theta +\mu ) & \frac{\beta \;(1-\phi )\;[S_{0}+(1-\varepsilon )]V_{0})}{K}\\ \kappa & -(\gamma +\mu +\delta ) \end{array}\right],$$

where the roots of the characteristic equation are the eigenvalues of the matrix *J*_2_(*E*_0_):

A 7 $$\lambda^{*2}+(\kappa +\theta +\mu )\;(\gamma +\mu +\delta )\;\lambda^{*}+(\kappa +\theta +\mu )\;(\gamma +\mu +\delta )-\frac{\kappa \;\beta \;[S_{0}+(1-\varepsilon )\;V_{0}]}{K}=0.$$

Equation (A 7) can be written in terms of $R_{0}$ as

A 8 $$\lambda^{*2}+(\kappa +\theta +\mu )\;(\gamma +\mu +\delta )\;\lambda^{*}+(\kappa +\theta +\mu )\;(\gamma +\mu +\delta )\;(1-R_{0})=0.$$

Now, using the Routh Hurwitz criterion [61], we can see that the solutions of the polynomial (A 8) have negative real parts if $R_{0}<1$, since (*κ* + *θ* + *μ*) (*γ* + *μ* + *δ*) > 0 and (*κ* + *θ* + *μ*)(*γ* + *μ* + *δ*) > 0. Hence, the DFE *E*_0_ is locally asymptotically stable when $R_{0}<1$. ▪

**A.3. Global stability of the disease-free equilibrium**

We develop a Lyapunov function to show that the DFE of system (2.2) is globally asymptotically stable. Constructing the Lyapunov function of the form

A 9 $$L(t)=\frac{\kappa }{\left(\kappa +\theta +\mu )(\gamma +\theta +\delta \right)}\;E(t)+\frac{1}{\left(\gamma +\theta +\delta \right)}\;I(t),$$

and differentiating (A 9) along the trajectory of the system, yields

A 10 $$L^{\prime }(t)=\frac{\kappa }{\left(\kappa +\theta +\mu )(\gamma +\theta +\delta \right)}E^{\prime }(t)+\frac{1}{\left(\gamma +\mu +\delta \right)}I^{\prime }(t).$$

Substituting for *E*^′^(*t*) and *I*^′^(*t*) in (A 10) gives

A 11 $$L^{\prime }(t)=\frac{\kappa }{\left(\kappa +\theta +\mu )(\gamma +\theta +\delta \right)}[\lambda S+(1-\varepsilon )\lambda V-(\kappa +\theta +\mu +\mu )E]$$

A 12 $$\;+\frac{1}{\left(\gamma +\mu +\delta \right)}\;[\kappa E-(\gamma +\mu +\delta )I].$$

On expanding (A 12), we have

A 13 $$L^{\prime }(t)=\frac{\kappa }{\left(\kappa +\theta +\mu )\;(\gamma +\mu +\delta \right)}(\lambda \;(S+(1-\varepsilon )V))-I.$$

Substituting for $\lambda =\frac{\beta (1-\phi )I}{K+I^{q}}$ in (A 13), yields

$$L^{\prime }(t)=\left(\;\frac{\kappa \;\beta \;(1-\phi )}{\left(\kappa +\theta +\mu )\;(\gamma +\mu +\delta )\;(K+I^{q}\right)}([S+(1-\varepsilon )V])-1\right)I.$$

It can then be easily shown that

$$\begin{array}{rl} L^{\prime }(t) & =\left(\;\frac{\kappa \;\beta \;(1-\phi )\;[S_{0}+(1-\varepsilon )\;V_{0}]}{(\kappa +\theta +\mu )\;(\gamma +\mu +\delta )\;K}-1\;\right)\;I\\ & \;-\frac{\kappa \;\beta \;(1-\phi )}{\left(\kappa +\theta )\;(\gamma +\mu +\delta \right)}\left[\frac{K((S_{0}-S)+(1-\varepsilon )\;(V_{0}-V))+I^{q}[S_{0}+(1-\varepsilon )\;V_{0}]}{\left(K+I^{q}\right)}\right]I, \end{array}$$

so that

A 14 $$L^{\prime }(t)\;\leq \;(\;R_{0}-1),$$

since *S*_0_ > *S* and *V*_0_ > *V*. Thus, *L*^′^(*t*) ≤ 1 if $R_{0}\;<\;1$, and equal to zero if *E* = *I* = 0.

**A.4. Bifurcation analysis**

The change in the qualitative characteristics of a solution as a control parameter is varied is known as a bifurcation. Bifurcation analysis is a powerful method for studying the dynamics of the steady state of nonlinear dynamical systems. At $R_{0}=1$, it implies that the bifurcation parameter $\beta =\hat{\beta }$, where

A 15 $$\hat{\beta }=\frac{K(\kappa +\theta +\mu )\;(\gamma +\mu +\delta )}{\kappa \;(s_{0}+(1-\varepsilon )v_{0})}=\frac{K\;\mu \;(\mu +\theta )(\kappa +\theta +\mu )\;(\gamma +\mu +\delta )}{k\;\Lambda \;(\mu +(1-\varepsilon )\;\theta )}.$$

Solving the Jacobian matrix $J(E_{0})$, (A 5), at $R_{0}=1$ gives a zero eigenvalue, and negative eigenvalues *λ** = −*μ*, − (*μ* + *θ*) twice and *λ** = −(*κ* + *θ* + *μ*)(*γ* + *μ* + *δ*).

Let *ω* = (*ω*_1_, *ω*_2_, *ω*_3_, *ω*_4_, *ω*_5_) and *ν* = (*ν*_1_, *ν*_2_, *ν*_3_, *ν*_4_, *ν*_5_) be the left and right eigenvectors of the matrix $J(E_{0})$ at $R_{0}=1$. To get the eigenvector *ω*, we multiply the matrix $J(E_{0})$ with *ω* and equate to zero, then solve for *ω*. This gives

A 16 $$\begin{array}{cc} & \omega_{1}=\frac{-\beta \;(1-\phi )\;S_{0}}{K\;(\mu +\theta )}\omega_{3};\;\omega_{2}=\frac{\left(\gamma +\mu +\delta \right)}{\kappa }\omega_{3};\;\omega_{4}=\frac{\gamma }{\left(\mu +\theta \right)}\;\omega_{3};\\ \;\mathrm{and}\; & \omega_{5}=\frac{1}{\mu \;(\mu +\theta )}\left[\frac{\theta ((\gamma +\mu +\delta )(\mu +\theta )+\gamma K)}{\kappa }-\frac{\beta \;(1-\phi )\;(S_{0}\theta +(1-\varepsilon )V_{0}(\mu +\theta ))}{K}\right]\omega_{3}. \end{array}\}$$

For the eigenvector *ν*, we multiply the transpose of $J(E_{0})$ with *ν* and equate it to zero, then solve for *ν* to get

$$\nu_{1}=\nu_{4}=\nu_{5}=0\;\text{and}\;\nu_{2}=\frac{\kappa }{\kappa +\theta +\mu }\;\nu_{3}.$$

The expression

$$f_{2}\;=\frac{\beta \;(1-\phi )\;x_{1}\;x_{3}}{K+x_{3}^{q}}+\frac{\beta \;(1-\phi )\;(1-\varepsilon )\;x_{3}\;x_{5}}{K+x_{3}^{q}}\;$$

is used to compute the coefficients *a* and *b*. The non-zero second partial derivatives of *f*_2_ at DFE are

$$\frac{\partial^{2}f_{2}}{\partial x_{1}\;\partial x_{3}}\;(E_{0})=\frac{\beta \;(1-\phi )\;}{K},\;\frac{\partial^{2}f_{2}}{\partial x_{3}\;\partial x_{5}}\;(E_{0})=\frac{\beta \;(1-\phi )\;(1-\varepsilon )}{K}.$$

Hence

$$\begin{array}{rl} a & =\nu_{2}\;\left(\omega_{1}\;\omega_{3}\;\frac{\partial^{2}f_{2}}{\partial x_{1}\;\partial x_{3}}\;(E_{0})+\omega_{3}\;\omega_{5}\frac{\partial^{2}f_{2}}{\partial x_{3}\;\partial x_{5}}\;(E_{0})\right)\\ & =\frac{\kappa }{\kappa +\theta +\mu }\;\nu_{3}\;\omega_{3}\;(\frac{-\beta \;(1-\phi )\;S_{0}}{K\;(\mu +\theta )}\cdot \frac{\beta \;(1-\phi )}{K}\;\omega_{3}+\frac{\beta \;(1-\phi )\;(1-\varepsilon )}{K}\;\omega_{3}\cdot \frac{1}{\mu (\mu +\theta )}\times \\ & \;\times \left(\frac{\theta \;((\gamma +\mu +\delta )(\mu +\theta )+\gamma \;k)}{K}-\frac{\beta \;(1-\phi )\;(S_{0}\;\theta +(1-\varepsilon )\;V_{0}\;(\mu +\theta ))}{K}\;\right)\;), \end{array}$$

which entails that

A 17 $$\begin{array}{rl} a & =\frac{\beta \;(1-\phi )\;\kappa }{K\;(\kappa +\theta +\mu )\;(\mu +\theta )}\;\nu_{3}\;\omega_{3}^{2}(\frac{\left(1-\varepsilon \right)}{\mu }\times \\ & \;\left(\frac{\theta \;(\gamma +\mu +\delta )\;(\mu +\theta )+\gamma \;\theta \;\kappa }{\kappa }-\frac{\beta \;(1-\phi )\;(S_{0}+(1-\varepsilon )\;V_{0}\;(\mu +\theta ))}{K}\right)-\frac{\beta \;(1-\phi )\;S_{0}}{K}). \end{array}$$

To obtain for *b*, we solve

$$\frac{\partial^{2}f_{2}}{\partial \beta \;\partial x_{3}}\;(E_{0})=\frac{S_{0}+(1-\varepsilon )\;V_{0}}{K},$$

so that

$$b=\nu_{3}\;\omega_{3}\;\frac{\partial^{2}f_{2}}{\partial \beta \;\partial x_{3}}\;(E_{0})\Rightarrow b=\;\nu_{3}\;\omega_{3}\;\frac{\left(S_{0}+(1-\varepsilon )\;V_{0}\right)}{K}\;>\;0,$$

since 0<ε<1.

Hence, the direction of the bifurcation depends on the value of *a*. If *a* < 0, it is a forward bifurcation and if *a* > 0, it is a backward bifurcation.

**A.5. Existence of an endemic state**

The EE point is the solution of the steady state when the disease, COVID-19, persists in the population. In this section, we prove the existence of an EE point for all possible assumed values of *q*; i.e. *q* = {0, 1, 2, 3}.

At the endemic equilibrium state *I* ≠ 0, *θ*(*t*) = *θ* and *β** = *β*(1 − *ϕ*)/(*K* + *I*^*q*^), we have

A 18 $$\begin{array}{cc} & S^{*}=\frac{\Lambda }{\beta^{*}I^{*}+\theta +\mu },\;E^{*}=\frac{(\gamma +\theta +\delta )I^{*}}{\kappa }\\ \;\mathrm{and}\; & R^{*}=\frac{\gamma I^{*}}{\mu +\theta },\;V^{*}=\frac{\theta (S^{*}+E^{*}+R^{*})}{(1-\varepsilon )\beta^{*}I^{*}+\mu }. \end{array}\}$$

Next, we consider different cases for the exponent *q*.

**A.6. Case *q* = 0**

When *q* = 0, $\beta^{*}=\frac{\beta (1-\phi )}{K}$. Substituting $\beta^{*}=\frac{\beta (1-\phi )}{K}$ into

A 19 $$\beta^{*}I^{*}S^{*}+(1-\varepsilon )\beta^{*}I^{*}V^{*}-(\mu +\kappa +\theta )E^{*}=0,$$

and solving for *I**, yields

A 20 $$A_{2}I^{*2}+A_{1}I^{*}+A_{0}=0,$$

where

A 21 $$\begin{array}{cc} & A_{2}=(1-\varepsilon )\beta^{2}{\left(1-\phi \right)}^{2}H,\\ & A_{1}=K(1-\varepsilon )(1-\phi )\beta (\mu +\theta )H+K\beta (1-\phi )\mu (\mu +\kappa +\theta )(\gamma +\mu +\delta )(\mu +\theta )\\ & \;-\Lambda (1-\varepsilon )\kappa \beta^{2}{\left(1-\phi \right)}^{2}(\mu +\theta )\\ \mathrm{and}\; & A_{0}=K^{2}\mu {\left(\mu +\theta \right)}^{2}(\mu +\kappa +\theta )(\gamma +\mu +\delta )[1-\frac{\beta (1-\phi )\beta \Lambda \kappa (\mu +(1-\varepsilon )\theta )}{K\mu (\mu +\theta )(\mu +\kappa +\theta )(\gamma +\mu +\delta )}], \end{array}\}$$

with

H=[μ(μ+κ+θ)(γ+μ+δ)+κ(θ(μ+δ)+μ(γ+μ+δ))].

By the definition of *R*_0_,

$$A_{0}=K^{2}\mu {\left(\mu +\theta \right)}^{2}(\mu +\kappa +\theta )(\gamma +\mu +\delta )[1-R_{0}].$$

**A.7. Case *q* = 1**

When *q* = 1, $\beta^{*}=\frac{\beta (1-\phi )}{K+I}$. Substituting $\beta^{*}=\frac{\beta (1-\phi )}{K+I}$ into equation (A 19) and solving for *I** gives

A 22 $$C_{2}I^{*2}+C_{1}I^{*}+C_{0}=0,$$

where

A 23 $$\begin{array}{cc} & C_{2}=\beta (1-\varepsilon )(1-\phi )[(1-\phi )\beta +\mu +\theta ]H\\ & \;+\mu (\mu +\theta )(\mu +\kappa +\theta )(\gamma +\mu +\delta )[(1-\phi )\beta +\mu +\theta ],\\ & C_{1}=K(1-\varepsilon )(1-\phi )\beta (\mu +\theta )H+K(1-\phi )\beta \mu (\mu +\kappa +\theta )(\gamma +\mu +\delta )(\mu +\theta )\\ & \;-\Lambda (1-\varepsilon )\kappa \beta^{2}{\left(1-\phi \right)}^{2}(\mu +\theta )+K^{2}\mu {\left(\mu +\theta \right)}^{2}(\mu +\kappa +\theta )(\gamma +\mu +\delta )(2-R_{0})\\ \mathrm{and}\; & C_{0}=K^{2}\mu {\left(\mu +\theta \right)}^{2}(\mu +\kappa +\theta )(\gamma +\mu +\delta )(1-R_{0}). \end{array}\}$$

**A.8. Case *q* = 2**

When *q* = 2, *β** = *β*(1 − *ϕ*)/(*K* + *I*^2^). Substituting *β** = *β*(1 − *ϕ*)/(*K* + *I*^2^) into equation (A 19) and solving for *I** gives

A 24 $$E_{4}I^{*4}+E_{3}I^{*3}+E_{2}I^{*2}+E_{1}I^{*}+E_{0}=0,$$

where

A 25 $$\begin{array}{cc} & E_{4}=\mu {\left(\mu +\theta \right)}^{2}(\mu +\kappa +\theta )(\gamma +\mu +\delta ),\\ & E_{3}=\beta (1-\varepsilon )(1-\phi )(\mu +\theta )H+\beta (1-\phi )\mu (\mu +\theta )(\mu +\kappa +\theta )(\gamma +\mu +\delta ),\\ & E_{2}=\beta^{2}{\left(1-\phi \right)}^{2}(1-\varepsilon )H+K\mu {\left(\mu +\theta \right)}^{2}(\mu +\kappa +\theta )(\gamma +\mu +\delta )(2-R_{0}),\\ & E_{1}=K(1-\varepsilon )(1-\phi )\beta (\mu +\theta )H+K(1-\phi )\beta \mu (\mu +\kappa +\theta )(\gamma +\mu +\delta )(\mu +\theta )\\ & \;-\Lambda (1-\varepsilon )\kappa \beta^{2}{\left(1-\phi \right)}^{2}(\mu +\theta )\\ \mathrm{and}\; & E_{0}=K^{2}\mu {\left(\mu +\theta \right)}^{2}(\mu +\kappa +\theta )(\gamma +\mu +\delta )(1-R_{0}). \end{array}\}$$

**A.9. Case *q* = 3**

When *q* = 3, *β** = *β*(1 − *ϕ*)/(*K* + *I*^3^). Substituting *β** = *β*(1 − *ϕ*)/(*K* + *I*^3^) into equation (A 19) and solving for *I** gives

A 26 $$G_{6}I^{*6}+G_{4}I^{*4}+G_{3}I^{*3}+G_{2}I^{*2}+G_{1}I^{*}+G_{0}=0,$$

where

A 27 $$\begin{array}{cc} & G_{6}=\mu {\left(\mu +\theta \right)}^{2}(\mu +\kappa +\theta )(\gamma +\mu +\delta ),\\ & G_{4}=\beta (1-\varepsilon )(1-\phi )(\mu +\theta )H,\\ & G_{3}=K\mu {\left(\mu +\theta \right)}^{2}(\mu +\kappa +\theta )(\gamma +\mu +\delta )(2-R_{0}),\\ & G_{2}=\beta^{2}{\left(1-\phi \right)}^{2}(1-\varepsilon )H,\\ & G_{1}=K(1-\varepsilon )(1-\phi )\beta (\mu +\theta )H+K(1-\phi )\beta \mu (\mu +\kappa +\theta )(\gamma +\mu +\delta )(\mu +\theta )\\ & \;-\Lambda (1-\varepsilon )\kappa \beta^{2}{\left(1-\phi \right)}^{2}(\mu +\theta )\\ \mathrm{and}\; & G_{0}=K^{2}\mu {\left(\mu +\theta \right)}^{2}(\mu +\kappa +\theta )(\gamma +\mu +\delta )(1-R_{0}). \end{array}\}$$

We study the existence of the possible number of endemic states by using the Descartes rule of sign, looking at the value of $R_{0}$. We study the possible number for each case of *q*. The results are summarized in tables 4–6.

**Table 4.** Number of possible positive real roots for cases *q* = 0, 1.

*A*_2_	*A*_1_	*A*_0_	range of *R*_0_	no. of possible positive real roots
+	*	−	*R*_0_ > 1	1
+	−	+	*R*_0_ < 1	0,2

*C*_2_	*C*_1_	*C*_0_	range of *R*_0_	no. of possible positive real roots
+	*	−	*R*_0_ > 1	1
+	−	+	*R*_0_ < 1	0,2

*Shows the possibility of all signs ( + , − , 0).

**Table 5.** Number of possible positive real roots for case *q* = 2.

*E*_4_	*E*_3_	*E*_2_	*E*_1_	*E*_0_	range of $R_{0}$	no. of possible positive real roots
+	+	+	*	−	$R_{0}>2$	1
+	+	−	−	−		
+	+	−	+	−		1,3
+	+	+	*	−	$1<R_{0}\leq 2$	1
+	+	+	−	+	$R_{0}<1$	0,2
+	+	+	+	+		0

**Table 6.** Number of possible positive real roots for *q* = 3.

*G*_6_	*G*_4_	*G*_3_	*G*_2_	*G*_1_	*G*_0_	range of $R_{0}$	no. of possible positive real roots
+	+	−	+	*	−	$R_{0}>2$	1,3
+	+	+	+	*	−	$1<R_{0}\leq 2$	1
+	+	+	+	−	+	$R_{0}<1$	0,2
+	+	+	+	+	+		0
